# Scavenger Receptors and Their Potential as Therapeutic Targets in the Treatment of Cardiovascular Disease

**DOI:** 10.4061/2010/646929

**Published:** 2010-08-17

**Authors:** Sam L. Stephen, Katie Freestone, Sarah Dunn, Michael W. Twigg, Shervanthi Homer-Vanniasinkam, John H. Walker, Stephen B. Wheatcroft, Sreenivasan Ponnambalam

**Affiliations:** ^1^Endothelial Cell Biology Unit, Institute of Molecular & Cellular Biology, LIGHT Laboratories, University of Leeds, Clarendon Way, Leeds LS2 9JT, UK; ^2^Leeds Vascular Institute, Leeds General Infirmary, Great George Street, Leeds LS1 3EX, UK; ^3^Academic Unit of Molecular and Vascular Medicine, Faculty of Medicine and Health, LIGHT Laboratories, University of Leeds, Clarendon Way, Leeds LS2 9JT, UK

## Abstract

Scavenger receptors act as membrane-bound and soluble proteins that bind to macromolecular complexes and pathogens. This diverse supergroup of proteins mediates binding to modified lipoprotein particles which regulate the initiation and progression of atherosclerotic plaques. In vascular tissues, scavenger receptors are implicated in regulating intracellular signaling, lipid accumulation, foam cell development, and cellular apoptosis or necrosis linked to the pathophysiology of atherosclerosis. One approach is using gene therapy to modulate scavenger receptor function in atherosclerosis. Ectopic expression of membrane-bound scavenger receptors using viral vectors can modify lipid profiles and reduce the incidence of atherosclerosis. Alternatively, expression of soluble scavenger receptors can also block plaque initiation and progression. Inhibition of scavenger receptor expression using a combined gene therapy and RNA interference strategy also holds promise for long-term therapy. Here we review our current understanding of the gene delivery by viral vectors to cells and tissues in gene therapy strategies and its application to the modulation of scavenger receptor function in atherosclerosis.

## 1. Introduction

Scavenger receptors comprise a structurally diverse group of proteins [1]. Originally identified by Brown and Goldstein, they were defined by their ability to bind modified forms of low density lipoprotein (LDL) including acetylated LDL (AcLDL) and oxidized LDL (OxLDL) and were thus implicated as key regulators in initiation and progression of atherosclerosis [2]. This family of proteins has expanded to include eight different classes of membrane and soluble proteins (Class A, B, C, D, E, F, G, and H) encoded by distinct and unrelated genes [3]. Scavenger receptor classes are grouped by the presence of shared structural domains; however there is great structural diversity between the different classes. Despite this lack of sequence similarity or identity, all scavenger receptors retain the capacity to bind modified lipid particles in addition to a diverse range of polyanionic ligands of host-derived or exogenous origins, for example, pathogens [4, 5].

## 2. Genetics of Scavenger Receptors

Class A scavenger receptors comprise at least four related genes: scavenger receptor A (SR-A), macrophage receptor with collagenous structure (MARCO), scavenger receptor with C-type lectin (SRCL), and scavenger receptor A-5 (SCARA5) [6–10]. The human and murine *SR-A* genes are located on chromosome 8 and can be transcribed to produce three (SR-AI/II/III) or two SR-A splice variants, respectively [11]. SR-AI/II is largely found on macrophages but are also present on endothelial cells and vascular smooth muscle cells (VSMCs). Oxidative stress, OxLDL, macrophage colony-stimulating factor (M-CSF), and phorbol esters can elevate SR-A levels [12–15]. SR-A is postulated to be proatherogenic due to its ability to mediate uptake of OxLDL in macrophages [16, 17]. Deficiency of SR-AI and SR-AII not only led to the formation of smaller atherosclerotic lesions, but also to a reduction of macrophage adhesion and increased susceptibility to bacteria and viruses [18, 19]. *MARCO* is located on human chromosome 2 or mouse chromosome 1 [20], and the gene product is expressed largely on macrophages and on splenic dendritic cells to a lower extent [9, 21]. MARCO is implicated in host defense and pathogen clearance since binding to dead or apoptotic cells, bacteria, and lipopolysaccharides elevates MARCO levels [22, 23]. When challenged with *Streptococcus pneumoniae*, wild type mice could clear the infection whereas the ability was impaired in MARCO^−/−^ mice, demonstrating the role of MARCO in the innate immune response against pathogens [24]. MARCO expression in human alveolar macrophages also plays a crucial role in the innate immunity against bacteria [25]. Human and murine *SRCL* genes are both located on chromosome 18 and can generate at least 2 splice variants in humans. In contrast to the other Class A gene products, SRCL is detected on endothelial cells but not macrophages [26] and may be involved in the innate immune response against fungal infections [27]. *SCARA5* is located on mouse chromosome 14: the resulting gene product is detected on epithelial cells but not macrophages [7] and may play unique role(s) in the innate immune system and atherosclerosis [28].

Class B contains at least four members: CD36, SR-B (also known as CLA-1 in humans), LIMPII-related genes, and CD163. *CD36* is located on human chromosome 7 or murine chromosome 5 [29] and its expression is mostly limited to cells of lymphoid and hematopoietic lineages including leukocytes, platelets, endothelial cells, adipocytes, VSMCs, and some epithelial cells; its levels are highest in macrophages [30, 31]. Double knockout SR-A^−/−^/CD36^−/−^ mice show increased foam cell formation and atherosclerotic lesion size, suggesting that CD36 acts as a major cellular receptor for OxLDL [16, 32, 33]. However, a different study using a triple knockout SR-A^−/−^/CD36^−/−^ApoE^−/−^ mouse demonstrated no change in atherosclerotic lesion size but decreased levels of various inflammatory gene products; ∼30% decrease in macrophage apoptosis and ∼50% decrease in plaque necrosis suggested delayed progression towards advanced, unstable atherosclerotic lesions [34]. In the nematode *C. elegans,* the CD36 orthologue (C03F11.3) mediates host defense against fungal pathogens [35]. Higher levels of a soluble form of CD36 are biomarkers of insulin resistance and plaque instability in patients with diabetes and internal carotid stenoses, respectively [36, 37]. CD36-deficient mice when challenged with pathogens were significantly more susceptible to the infections [38, 39]. Humans expressing CD36 allelic variants were also more susceptible to malaria [40] demonstrating its important role in the immune system. *SR-BI* (*SCARB1*) is located on human chromosome 12 or mouse chromosome 5 [41] and encodes two protein isoforms (SR-BI/II) [42] in monocytes, macrophages, hepatocytes, and adipose and steroidogenic tissues [43]. SR-BI expression is elevated by either PPAR*α*, PPAR*γ*, testosterone, PUFA, or TSA [44–47] and downregulated by either OxLDL, TNF-*α*, IL-1, or lipopolysaccharides [48, 49]. SR-B1 is a receptor for hepatitis C virus, *Plasmodium,* and mycobacteria pathogens [50–52]. In contrast to other scavenger receptors, SR-BI could provide protective function(s) against atherosclerosis by increasing the macrophage-based cholesterol efflux into HDL particles followed by liver HDL clearance and excretion [47, 53–56]. LIMPII is located on human chromosome 4 or mouse chromosome 5 and has a similar expression profile to SR-BI [31]. *CD163 (M130) *[57] is located in human chromosome 12 [58] and expressed in monocytes and macrophages in both membrane-bound and soluble forms [59] where it plays an important role in the regulation of anti-inflammatory responses, pathogen recognition, and atheroprotection probably through elevation in expression of heme oxygenase and in removing free hemoglobin [60–63]. Patients with hematological, inflammatory, and lysosomal storage diseases have also a high level of soluble CD163, and it may thus serve as a biomarker for such conditions [64, 65].

Class C comprises of just one scavenger receptor, dSR-C1 which has only been so far identified in the fruit fly *Drosophila melanogaster*. dSR-C1 is a pattern recognition receptor for bacteria expressed in hemocytes and macrophages during fly embryonic development [66]. It can recognize bacteria and may play a role in the innate immune system of the insect [67].

Class D comprises the CD68 and lysosomal membrane glycoprotein (Lamp) gene products. *CD68* is located on human chromosome 17 and the murine orthologue (also called macrosialin) is located on murine chromosome 11 [68]. Macrophages, Langerhans cells, dendritic cells, and osteoclasts express CD68 in pattern similar to the class B gene products [69]. The expression levels can be elevated by OxLDL, GM-CSF, and phorbol ester but inhibited by TNF-*α* and lipopolysaccharides [70–72]. Macrosialin levels are also observed to be increased by a proatherogenic diet: OxLDL and macrosialin were both found in macrophages within atherosclerotic plaques from ApoE-deficient mice [73]. Macrosialin has been identified as a receptor for OxLDL [74–76] although this view has been challenged [77]. The three *Lamp *genes (1, 2, and 3) are located in human chromosome 13, X, and 3 or murine chromosomes 8, X, and 16, respectively [78]. Lamp-1 and -2 are constitutively and widely expressed whereas Lamp-3 is elevated during dendritic cell maturation implying a functional link to the immune system [79].

Class E comprises of just one member: the lectin-like oxidized low density lipoprotein receptor 1 (LOX-1). *LOX-1* (*OLR1*) is located on human chromosome 12 [80] or mouse chromosome 6 and is expressed on endothelial cells, macrophages, smooth muscle cells, and platelets [81, 82]. The resting levels are relatively low but elevated by proinflammatory stimuli including OxLDL, inflammatory cytokines, for example, TNF-*α*, shear stress, oxidative stress, phorbol ester, endothelin-1, and angiotensin II [83–87]. A splice variant (LOXIN) conferred protection against the proatherogenic LOX-1 effects by forming inactive heterodimers with LOX-1 and blocking OxLDL-induced apoptosis in macrophages [88, 89]. A human LOX-1 allelic polymorphism (K167N) is postulated to increase the risk of CVD in a patient cohort [90]. However, further investigations into the associations between the LOX-1-K167N polymorphism, myocardial infarction (MI) and cardiovascular disease (CVD) have produced conflicting data [91, 92] suggesting that this polymorphism has no effects on CVD incidence [93]. The expression profile of a soluble LOX-1 species was elevated in obese postmenopausal women [94], and it is a biomarker for type 2 diabetes mellitus and atherogenesis [95–97]. In dendritic cells, LOX-1 can act as a receptor that mediates the uptake of antigens [98]. Overexpression of LOX-1 in CHO cells led to bacterial binding and uptake [99]. Macrophage LOX-1 depletion inhibits foam cell formation suggesting a role in atherosclerotic plaque initiation and progression [100]. Importantly, the incidence of atherosclerotic plaques is significantly lowered in LOX-1-deficient mice [101]. 

Class F consists of the SREC gene products (scavenger receptors expressed by endothelial cells) which are expressed on mammalian endothelial cells and macrophages [102] and also in nematodes [103]. The *SREC-I* gene is related to the EGF precursor gene [104] and is located on human chromosome 17 but the murine orthologue called *SCARF-1* is located on mouse chromosome 11. In humans, alternative splicing gives rise to at least five different membrane-bound and soluble protein isoforms [105]. SREC-I levels are elevated by lipopolysaccharides [102] and repressed by cytokines such as IL-1*α*, IL-1*β*, and TNF-*α* [105]. In humans, another gene called *SREC-II* that displays ∼35% similarity to *SREC-I* is located on chromosome 22 [104, 106]. Murine *SREC-II* is located on chromosome 16 [107]. In *C. elegans*, a SREC-like gene product called CED-1 is implicated in the engulfment of apoptotic cells during animal development and immune defense against pathogens [35, 108, 109]. SREC-I is a receptor for Ac-LDL [102].

The chemokine ligand CXCL16 is a class G scavenger receptor that binds phosphatidylserine and oxidized lipoprotein (SR-PSOX). *SR-PSOX* is located on human chromosome 17 and mouse chromosome 11. SR-PSOX is highly expressed on macrophages, smooth muscle cells, dendritic cells, kidney and B cells with lower levels detected on the endothelium, and T cells [110–119]. Monocyte SR-PSOX expression is increased by TNF-*α*, IFN-*γ*, LPS, or OxLDL stimulation [113, 115, 120]. In addition to SR-PSOX links to atherosclerosis [120, 121] where the molecule was induced *in vitro* and *in vivo* by atherosclerosis-promoting inflammatory signals [122], it is also involved in acute and adaptive experimental autoimmune encephalomyelitis [123], CD8+ T cell recruitment during inflammatory valvular heart disease [124], and bacterial phagocytosis [116]. A soluble form of SR-PSOX functions as an activated T cell and NK cell-recruiting chemokine [112, 125] and is a biomarker for acute coronary syndrome [126].

Class H scavenger receptors consist of Fasciclin, EGF-like, lamin type EGF-like and link domain-containing scavenger receptor-1 (FEEL-1), also known as stabilin-1 or CLEVER-1 [127] and FEEL-2 (stabilin-2) a paralogous protein with 39% sequence identity to FEEL-1 [128–130]. The *FEEL-1* gene (*STAB1*) is located on human chromosome 3 and mouse chromosome 14, and the *FEEL-2* gene (*STAB2*) is located on human chromosome 12 and murine chromosome 10. Expression levels of both FEEL-1 and -2 are high in the liver and lymph nodes. FEEL-1 is expressed on monocytes, macrophages, and endothelial cells whereas FEEL-2 expression was not detected on these cell types in humans [128]. FEEL-2 was found to be expressed in sinusoidal endothelial cells in the liver, lymph node, spleen, and bone marrow in mice as well as heart valve mesenchyme, brain, eyes, and kidneys [131], with expression levels being increased during development in the zebrafish *Danio rerio* [132]. FEEL-1 is known to undergo alternate splicing to yield an isoform lacking exon 27 [130]. Sorting nexin 17 (SNX17) is required for maximum cell surface expression of FEEL-1 [133]. Knockdown of SNX17 leads to a dramatic reduction in cell surface expression, due to increased degradation of the receptor.

## 3. Scavenger Receptor Structure and Function

Scavenger receptors are present on different tissues ranging between macrophages, monocytes, platelets, endothelial, smooth muscle, and epithelial cells. In addition to vascular tissues, they are also detected in adipose and steroidogenic tissues (Table 1) [9, 13, 82, 110, 134–137]. A general mechanism underlying scavenger receptor levels is the elevation of gene expression in response to ligand binding to cell surface receptors, thus generating a positive feedback loop that mediates enhanced ligand clearance and/or accumulation [138, 139]. This is in contrast to other membrane-bound receptors such as the low-density lipoprotein receptor (LDL-R) that is downregulated in response to binding LDL ligand, thus exhibiting a negative feedback mechanism [140].

**Figure 1:**
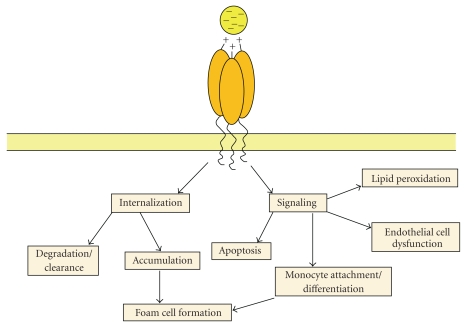
*A generic model for scavenger receptor-mediated ligand binding, internalization, and signal cascade activation. *Scavenger receptors bind negatively charged ligands through clusters of conserved positively charged residues. Ligands are internalized by scavenger receptors using a range of different clathrin-dependent and independent pathways. Ligands can be degraded or accumulate. Ligand binding can activate signaling cascades leading to diverse cellular functions including lipid peroxidation, apoptosis, endothelial cell dysfunction, and monocyte attachment and differentiation leading to foam cell formation.

Scavenger receptors are generally classified as membrane-bound proteins that bind modified LDL particles and other polyanionic ligands. These include AcLDL, OxLDL, Gram-positive and Gram-negative bacteria, apoptotic cells, *β*-amyloid fibrils, and advanced glycation end products (AGE) (Table 1) [4, 5]. Following ligand binding, scavenger receptors can mediate intracellular signaling and/or ligand internalization. A generic model for such regulation is outlined in Figure 1. Although there is little structural homology between the ligand-binding domains of scavenger receptors from different classes, mutagenesis studies have revealed some conserved characteristics. These include positively charged arginine or lysine clusters in the ligand-binding domain of either the LOX-1 scavenger receptor [141] or CD36 [142], respectively. Such amino acid clusters appear to be required to mediate electrostatic interactions with the predominantly negatively charged modified lipid particle or polyanionic ligand although other noncharged hydrophilic residues may also be involved [141]. The avidity of ligand binding is also enhanced through the formation of scavenger receptor dimers [143], trimers [144], and higher-order oligomers [145].

Scavenger receptor-ligand complexes can undergo receptor-mediated endocytosis, trafficking through the endosome-lysosome system leading to degradation or accumulation of ligand. Different mechanisms of endocytosis have been postulated for the individual classes of scavenger receptors including clathrin-dependent [146], clathrin-independent [147], and lipid raft-mediated [148] events. This diversity in scavenger receptor endocytosis is not surprising considering the sequence diversity and different endocytic motifs within the cytoplasmic domains of the different scavenger receptors [146, 147]. Following endocytosis and delivery to endosomes, it is likely that many scavenger receptors are recycled back to the plasma membrane where they can mediate further ligand binding, clearance, or accumulation.

Ligand binding to scavenger receptors activates intracellular signaling cascades leading to diverse physiological outputs including apoptosis, endothelial cell dysfunction, and lipid peroxidation. One aspect of scavenger receptor activation is monocyte infiltration and differentiation leading to foam cell formation, a key event in atherosclerotic plaque initiation and progression. For example, activation of the Class B CD36 scavenger receptor is linked to phosphorylation and activation of c-Src and MAP kinase pathway thus triggering macrophage differentiation into foam cells [149]. Another model is the LOX-1 scavenger receptor where ligand binding stimulates reactive oxygen species (ROS) production, both MAPK and NF-*κ*B activation leading to increased expression of different adhesion gene products. Such elevated expression in endothelium can enable monocyte infiltration, ultimately leading to monocyte differentiation and foam cell formation.

## 4. Current Atherosclerosis Therapies

Atherosclerosis is a leading cause of mortality in Europe and Western countries [150]. The subversion of human vascular function by atherosclerosis can lead to cardiovascular morbidity and mortality, including ischemic stroke, ischemic heart disease, myocardial infarction, and peripheral arterial disease. The causes of atherosclerosis are multifactorial, meaning that single intervention therapy has as yet not succeeded in major reductions in disease incidence. Ongoing large investments by many countries worldwide are directed towards the prevention of cardiovascular disease by modifying environmental risk factors. Within the United Kingdom alone, a vascular risk and assessment program is currently in its initial roll out phase, aiming to tackle modifiable risk factors in a healthy 40–74-year age group (http://www.dh.gov.uk/en/Publicationsandstatistics/Publications/PublicationsPolicyAndGuidance/DH_083822). Despite economic modeling predicting a relatively large annual cost (US$60 million), this approach is predicted to prevent approximately 9500 cases of myocardial infraction and strokes annually. This would thus also be cost effective in the long term. (http://www.dh.gov.uk/en/Publicationsandstatistics/Publications/PublicationsPolicyAndGuidance/DH_085869).

Pharmacological agents have only been partially successful in attenuating the clinical manifestations of atherosclerosis with the most dramatic effects achieved by statins, which inhibit 3-hydroxy-3-methyl glutaryl coenzyme A (HMG-CoA) reductase. Statin therapy has reduced the 5-year incidence of major cardiovascular events by ∼20% with each millimole per liter reduction in levels of cholesterol in LDL particles [151]. It is thought that statins exert their effect through inhibiting the rate-limiting step in cholesterol biosynthesis, which in turn leads to elevation of LDL receptor expression. However there is much debate over the importance of additional lipid-independent modes of action including anti-inflammatory effects [152]. Clinical treatment of established atherosclerotic plaques is becoming more technologically advanced, with routine intra-arterial catheterization and angioplasty of damaged arterial blood vessels. Stents are commonly used, and new technologies, such as drug-eluting stents, raise exciting possibilities for potential gene therapy as well. Evaluation of a patient's genetic background in atherosclerotic plaque initiation and progression could be essential to provide major disease alleviation by combining intervention, medication, and lifestyle modification in tailored therapies. Targeting the scavenger receptor gene products that mediate the response to and/or uptake of modified LDL holds great promise in the prevention of cardiovascular disease.

## 5. Gene Therapy

Gene therapy is the process of ameliorating or curing a genetic disease by introducing a fragment of genetic material into diseased or dysfunctional cells. Viral or nonviral vectors are the vehicles used to transfer and express specific genes within a target cell and thus used to correct genetic disorders. The idea of using genetic material to treat human diseases gained prominence in 1960s [153], and in 1973, the first attempt used a wild-type human papilloma virus in attempting to correct hyperargininaemia [154] but this was unsuccessful. Even though subsequent gene therapy attempts were controversial [155], this area of biomedicine obtained the first signs of success by tackling adenosine deaminase-deficient severe combined immunodeficiency (ADA-SCID) [156]. The first ADA-SCID patient treated successfully using this technology has subsequently led a relatively normal lifestyle with amelioration of the disease symptoms [157]. By 2007, >1300 gene therapy clinical trials have taken place of which the majority of trials (67%) aimed at cancer treatment; gene therapy of cardiovascular diseases formed the second largest cohort of clinical trials (9%). The number of clinical gene therapy trials currently exceeds 1500 (http://www.wiley.co.uk/genmed/clinical/) and may well live up to the expectations of becoming the “twenty-first century medicine” to deliver personalized healthcare [158].

Nevertheless, successful clinical gene therapy has encountered numerous problems. The two major problems that usually hamper gene therapy efficacy are (1) immune response(s) against the protein products of the transgene or the vector and (2) insertional mutagenesis by the viral vector. Since viral vectors are based on pathogenic viruses, they can induce immune responses [159–161], and much of the human population may have preexisting immunity against human viruses. Depending on the conditions used, this immunogenicity can lead to adverse effects. During one study on adenoviral-mediated treatment of ornithine transcarbamylase (OTC) deficiency, one of the eighteen subjects died as a result of an exacerbated immune response to the injected adenoviral vector carrying an *E1-E14* deletion [162, 163]. Leukemia induction was also noticed in a mouse model following gene transfer using retroviral vectors caused by vector integration into the ecotropic viral integration *Site-1* (*Evi1*) [164]. Two different gene therapy studies on X-linked SCID (SCID-X1) using retroviral vector integration resulted in leukemia induction in four out of nine subjects with one death [165, 166] and leukemia in one out of ten subjects [167, 168]. Retroviruses and lentiviruses undergo obligatory integration of the provirus into the host genome as a part of the life cycle, and this may lead to activation or increased expression of nearby host genes [169, 170]. In the case of retro- and lentiviral vectors, the promoter enhancer elements located in the viral termini appear responsible for this altered host gene expression profile [171, 172].

In contrast, although the viral termini located within adeno-associated viruses [173, 174] and adenoviruses [175–177] have enhancer and promoter activities, insertional mutagenesis caused by vectors based on these viruses have not as yet been reported. However, even in the case of adenoviral vectors which have been perceived as nonintegrating vectors, there are instances of viral integration into the chromosomal DNA close to or within host genes and instances of genetic mutations and rearrangements [178, 179]. A slightly higher percentage of adeno-associated viral vector DNA [180, 181] integrated into genes causing chromosomal deletions and translocation [182–184]. Despite the lack of adverse affects in cystic fibrosis gene therapy trials, the effects caused by administration of different viral and nonviral gene therapy vectors were not sufficient to cause regression of clinical symptoms; natural lung adaptation to alien particle administration may have also hindered patient gene transfer [185]. However, successful gene therapy in the treatment of ADA-SCID and lack of adverse effects [186] holds much promise for further work.

## 6. Gene Therapy Vectors

Gene therapy vectors are genetic vehicles used to transfer DNA sequences or specific genes from the laboratory bench into the diseased cells or tissues and are viral or nonviral in origin. Viruses are well adapted to infect cells or tissues, and these adaptations have been utilized to generate viral vectors for gene therapy. Such viral vectors constitute 66% of clinical gene therapy trials worldwide (http://www.wiley.co.uk/genmed/clinical/). Both viral and nonviral vectors have their advantages and disadvantages in gene therapy (summarized in Table 2).

### 6.1. Nonviral Gene Therapy Vectors

Nonviral gene delivery systems utilize physical force or chemical methods to deliver the genetic material to the cell. Major nonviral vectors used in gene therapy include circular plasmid or linear DNA complexed with nanoparticles [187, 188] or liposomes (cationic lipid-DNA complex) [189, 190]. Plasmid DNA can be transferred into the cells using a gene gun [191] where the DNA is bound to high density particles like gold and transferred at high velocities into the cell [192] or by electroporation [193], using transposable elements [194] or DNA:RNA oligonucleotide hybrids [195]. Even though nonviral gene therapy methods are used in clinical trials [196] they usually exhibit lower gene transfer efficiency and transient gene expression [197–200]. This is especially true when both viral and nonviral systems were compared simultaneously [201]; the immune response [202–205] may also limit the therapeutic capability of nonviral gene therapy.

### 6.2. Viral Gene Therapy Vectors

Since viruses are well adapted to evade the host immune responses and to deliver the genetic material into the host cells, gene therapy vectors based on viruses have been more effective so far and currently account for two-thirds of all gene therapy clinical trials worldwide (http://www.wiley.co.uk/genmed/clinical/). Barring the use of vectors in suicide gene therapy [206], viral gene delivery systems utilize viral vectors with defective replication capabilities. The coding region of the viral genome is replaced by foreign genetic material, leaving only the *cis*-acting elements essential for viral packaging and/or integrating into the host genome on the vector. Producer cell lines can provide the essential viral gene products either totally by themselves or by the assistance of other systems, which are used to generate nonreplicating viral vectors [207]. Currently, vectors based on adeno-associated viruses, retroviruses, and adenoviruses form the majority of the viral vectors used as gene delivery systems.

#### 6.2.1. Adeno-Associated Viral (AAV) Vectors

Adeno-associated viruses belong to the *Parvoviridae* family which have a nonenveloped icosahedral capsid containing a single-stranded DNA genome. This viral DNA has *cis*-acting palindromic inverted terminal repeats at each end which form hairpins that are essential for DNA replication and packaging [208]. Most of the current AAV-based gene therapy vectors are derived from AAV-2 subtype. This virus is dependent on coexpression of an adenovirus or herpes helper virus for gene products essential for lytic productive infection where the genome is replicated, and virions are produced. In the absence of the helper virus, AAV-2 undergoes site-specific integration to establish a latent state. The provision in *trans* of the AAV Rep (regulatory) and Cap (structural capsid) genes together with the adenoviral early viral genes (provided by a helper virus) is needed to generate AAV vectors for gene therapy [158, 207, 209]. Despite the smaller AAV transgene capacity [210] and the preexisting immunity against AAV [211], these vectors have been used successfully in animal models of retinal disorders [212, 213], cystic fibrosis [214, 215], hemophilia B [216, 217], muscular dystrophy, and DNA vaccination [218]. They are currently used in human clinical gene therapy trials [219].

#### 6.2.2. Adenoviral Vectors

Adenoviruses belong to the *Adenoviridae *family and contain nonenveloped icosahedral capsid with a double-stranded DNA genome. A *cis*-acting inverted terminal repeat is present at each end of the DNA and a packaging signal at the 5′ terminus [220]. Some of the early adenoviral genes (e.g., *E1*) have transforming and transactivating functions and were thus replaced by inserted DNA sequences or the gene of interest in the first generation adenoviral vectors. However, this did not prevent low level expression of other adenoviral gene products, including those with immunogenic and toxic properties causing rapid clearance from host *in vivo * [221, 222]. To both avoid this adaptive immune response and to increase viral transgene capacity, high capacity adenoviral vectors (HC-AdV) were developed where the only viral elements present are the* cis-*acting ITRs and packaging signal with viral gene products needed for replication provided in* trans* by a packaging-deficient helper virus [223]. Even though the innate immunity against adenoviral capsid would still elicit an immune response [161], this may be circumnavigated either by using adenoviral vectors of different serotypes [224] or by modifications with synthetic polymers [225]. The large cloning capacity of 36 kb and the longevity of transgene expression [226–228] in tissues with low cellular turnover hint at potentially successful gene therapy. Adenoviral vectors have been used successfully in rodent, canine, and primate models of cardiovascular diseases [229, 230], muscular dystrophy [231, 232], glycogen storage diseases [233], hemophilia [234], cancer [235–237], retinal disorders [238], and DNA vaccination studies [239]. Adenoviral vectors comprise the largest group of the largest group of vectors (24%) used in clinical gene therapy trials (http://www.wiley.co.uk/genmed/clinical/) [240, 241].

#### 6.2.3. Retro- and Lentiviral Vectors

Retroviruses are enveloped single-stranded RNA viruses where the RNA genome is reverse transcribed into a DNA provirus which then integrates into the host chromosomal DNA during its life cycle. The viral genome is flanked by long terminal repeats (LTRs) which along with the packaging signal and a truncated *gag* gene comprising the *cis*-acting elements essential for functionality. Retroviral genes encoding the capsid proteins, the viral protease, the reverse transcriptase, and the integrase are supplied in *trans* by transient transfection of plasmids to generate an assembled virus with gene delivery capability [242–244]. Lentiviruses are more complex, regulatory and accessory genes, and have the capability to infect dividing and nondividing cells in contrast to retroviruses which can only infect dividing cells. Functional lentiviral vectors also need expression of the Rev (cytoplasmic transport of the RNA) and the Tat (viral promoter transactivator) viral gene products.

One biosaftey issue when using viral gene therapy is that extensive genetic recombination of the viral and host genomes could generate replication-competent viruses. Whilst the generation of replication-competent adenoviruses may cause only relatively mild health problems, this is likely to be dangerous if replication-competent lentiviruses are generated. To reduce this risk during the packaging process *in vitro *using cultured cell lines, *gag *and *pol *(with additional *tat* and *rev *for lentiviral vectors) and *env* are present on different plasmids. A further biosafety improvement is removal of the U3 region (enhancer-promoter) of the viral LTR to generate self-inactivating (SIN) vectors [245]. Since reverse transcription means that both the 5′ and 3′ U3 regions of the provirus DNA are transcribed, this deletion would abrogate synthesis of a complete RNA viral genome packaged into virions. One feature of the retrovirus life cycle is integration of the provirus into the host genome causing persistent gene expression [246]. Gene therapy using retro- or lentiviral vectors has been successfully used in the gene therapy experiments for treating Duchenne muscular dystrophy [247], hemophilia [248], Fanconi anemia [249], diseases of the central nervous system [250], DNA vaccination [251], X-linked SCID, adenosine deaminase SCID, and chronic granulomatous disease [168, 252]. These successes of these studies in a combination of rodent, canine, primate models have now led to ∼21% of current human clinical trials for gene therapy.

## 7. Scavenger Receptor Gene Therapy

Even in the late 1980s, cardiovascular dysfunction was a major focus of gene therapy trials. In a rabbit model of homozygous familial hypercholesterolemia, a retroviral LTR promoter was used to overexpress human LDL-R in fibroblasts [253]. Elsewhere, retroviral vectors containing *LacZ *encoding *β*-galactosidase were used for *ex vivo* transduction of canine and porcine endothelial cells: *β*-galactosidase expression could be detected after surgical implantation into canine and porcine models [254, 255]. Currently, the proportion of clinical gene therapy trials for cancer (65%) is followed by the second largest trial group on cardiovascular disease (9%) (http://www.wiley.co.uk/genmed/clinical/).

As mentioned earlier, scavenger receptor function is associated with both healthy and pathophysiological processes ranging between homeostasis, apoptotic cell clearance, diabetic necropathy, age-induced cardiomyopathy, and antigen cross-presentation in Alzheimer's disease [256–260]. Importantly scavenger receptor function is heavily implicated in atherosclerotic plaque initiation and progression [261], making this diverse protein supergroup [1] an attractive target for gene therapy (Figure 3). Currently, the majority of scavenger receptor gene therapy studies have utilized adenoviral vectors (Table 3).

### 7.1. LOX-1 Gene Therapy

A mouse knockout model lacking LOX-1 suggested that this was a key contributory factor in driving lipid accumulation in vascular tissues [101], raising the question as to whether these properties could be manipulated using gene therapy. A first-generation adenoviral vector expressing human LOX-1 was used to successfully express LOX-1 transiently in vascular smooth muscle cells (VSMCs) and other cells [262]. A first-generation first-generation adenoviral vector was used to provide ectopic expression of LOX-1 in hepatic tissues of the ApoE-deficient mice, leading to increased OxLDL excretion, reduction in plasma OxLDL, and complete loss of atherosclerotic plaque initiation and progression [263]. In addition, oxidative stress and inflammatory responses were reduced in the mice infected with adenovirus LOX-1. Nevertheless, OxLDL levels returned to control baseline levels 3 weeks after hepatic LOX-1 overexpression. This profile of gene expression correlates with the expected duration of transgene expression using first generation adenoviral vectors [221]. This raises the question whether more sustained long-term LOX-1 expression using stable ectopic expression systems or integrating viruses would be a better strategy to inhibit atherosclerosis.

### 7.2. SR-A Gene Therapy

A retroviral vector with a bovine SR-AII cDNA transgene was used to show increased lipid accumulation, foam cell formation, and predisposition to apoptosis in fibroblasts and smooth muscle cell lines [264]. This suggests that manipulation of SR-A levels might be advantageous in hindering proatherogenic responses in vascular tissues [264]. A hybrid gene containing the human CD68 promoter upstream of truncated human *SR-AI* encoding the extracellular domain alone and expressed using an adenoviral vector inhibited degradation of AcLDL and OxLDL particles and subsequent foam cell formation [265]. One conclusion is that soluble SR-AI binds to modified LDL particles and sequesters such ligands away from the wild-type membrane-bound scavenger receptors.

Using the LDL-R knockout mouse that develops atherosclerotic lesions, overexpression of soluble human SR-AI using this adenoviral system completely blocked plaque initiation and progression [266]. However, similar to gene expression profiles for first- and second-generation adenoviral vectors, the plasma soluble SR-AI returned to control baseline levels after 4 weeks [266]. When adeno-associated viral vectors were used to express soluble SR-AI in the same mouse model, the atherosclerotic lesion area was reduced and persistence of soluble SR-AI plasma levels was observed for 6 months [267]. Expression of murine MARCO using a lentiviral vector in cultured cells suggests that quality control along the secretory pathway is essential for scavenger receptor assembly and presentation at the plasma membrane [274]. SR-A may also be a receptor for adenovirus binding and host cell entry [275], and this could be further exploited to block macrophage lipid accumulation leading to foam cell formation during atherosclerosis.

### 7.3. SR-BI Gene Therapy

High-density lipoprotein (HDL) particles can mediate reverse cholesterol transport, have antiatherogenic properties, and are recognized by the SR-BI glycoprotein [276, 277]. Current gene therapy used first-generation adenoviral vectors to express murine SR-BI. Transient hepatic expression of murine SR-BI in mice increased HDL clearance, reduction in plasma HDL levels, and increased biliary cholesterol levels [54]. These effects are either due to increased hepatic uptake of HDL and/or the increased cholesterol secretion into the bile. Similar results were obtained with the same adenoviral vector delivered into LDL-R knockout mice with reduction of both early and advanced atherosclerotic lesions [55]. One explanation is that SR-BI overexpression resulted in a reduction of all three of HDL, LDL, and VLDL levels [278]. However, in human ApoB transgenic mice, SR-BI expression from the same vector resulted in a much lesser LDL-metabolism compared to HDL metabolism [279]. A xenogenic model comprising *ApoAI* knockout mice and SR-BI overexpression was used to examine transplanted human HDL processing. Here, small and dense HDL particles are not cleared from the circulation but remodel in the plasma to form larger HDL particles [280]. Similar SR-BI expression in a rabbit model caused reduction in HDL levels and increased LDL levels [268]. Again, SR-BI overexpression increased biliary excretion of cholesterol [269].

HDL binding to SR-BI can activate intracellular signaling leading to increased endothelial nitric oxide synthase (eNOS) activity [281]. Coexpression of SR-BI and Apobec 1 (essential in ApoB mRNA editing, resulting in the truncated ApoB48 product) in immortalized hepatic cells using a HC-Ad vector caused reduction in ApoB levels. Using the same system on immortalized endothelial cells caused increased eNOS phosphorylation and elevated nitric oxide levels [270]. However, the amount of HDL normally absorbed by the liver from the bloodstream is relatively low [282]. *SR-BI* allelic polymorphisms also do not correlate with variations in plasma HDL levels [283]. More clinical gene therapy studies are desirable to fully test whether this molecule is a good candidate for alleviating atherosclerosis. 

### 7.4. CD36 Gene Therapy

CD36 is another Class B scavenger receptor that can mediate oxidized LDL binding and internalization [284]. Liver overexpression of CD36 using a first-generation adenoviral vector significantly increased cellular fatty acid uptake including hepatic fatty acid, plasma, and hepatic triglycerides [273]. Constitutive expression of murine SR-BI or the murine CD36 using a first-generation adenoviral vector in cultured cells *in vitro* showed that SR-BI-mediated uptake of cholesterol esters was higher than CD36 [271]. Hepatic SR-BI overexpression significantly reduced HDL levels whereas CD36 overexpression had little effect [271]. However, in another study using the same *in vitro* model, CD36-mediated OxLDL internalization resulted in significantly higher lipid particle degradation, compared to SR-BI [272]. These findings suggest significant differences between the two proteins within the same class and that CD36 overexpression may be more beneficial by promoting the clearance of modified lipid particles.

### 7.5. SR-PSOX Gene Therapy

The scavenger receptor that binds to phosphatidylserine and oxidized lipoprotein (SR-PSOX) is highly expressed within atherosclerotic lesions [111], and elevated expression in macrophages stimulates OxLDL uptake [285]. Reduction of human SR-PSOX levels in immortalized monocytes by RNAi using a lentiviral vector decreased lipid accumulation and foam cell development [120]. However, in double knockout mice lacking both LDL-R and SR-PSOX, there was accelerated atherosclerosis with increased macrophage recruitment to the aortic arch [121]. The potential role of SR-PSOX in the innate immune system to mediate pathogen clearance [125, 286] suggests that more studies may be needed to ascertain potential benefits of the SR-PSOX manipulation *in vivo.*

## 8. Conclusions

**Figure 2:**
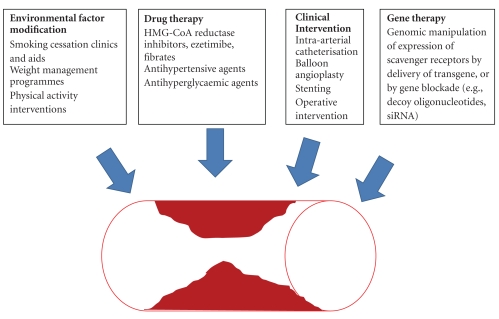
*Treatment of atherosclerosis.* A schematic to display the potential synergistic role of gene therapy in the treatment of atherosclerosis.

**Figure 3:**
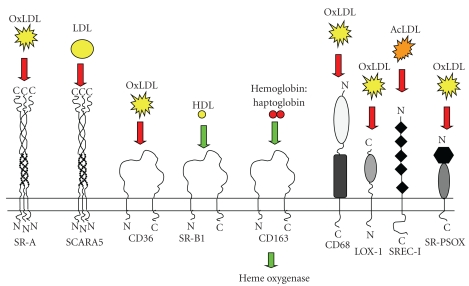
*Major scavenger receptors and their effects on atherosclerosis*. A schematic showing the role of major scavenger receptors in atherosclerosis. Red arrows indicate proatherogenic effects; green arrows indicate antiatherogenic or protective effects. SR-A (scavenger receptor class A) mediates uptake of OxLDL (oxidised low density lipoprotein) in macrophages; SCARA5 (scavenger receptor A5) down-regulation results in reduction of aortic LDL (low density lipoprotein) deposition; CD36 is probably a receptor of OxLDL; SR-B (scavenger receptor B) increases cholesterol efflux; CD163 exerts its protective actions through elevation of IL-10 and heme oxygenase; CD68 is a possible receptor for OxLDL; LOX-1 is a receptor for OxLDL; SREC-1 (scavenger receptors expressed by endothelial cells) is a receptor for AcLDL (acetylated low density lipoprotein); SR-PSOX (scavenger receptor that binds to phosphatidylserine and oxidized lipoprotein) binds to OxLDL (figure adapted from [1]).

Treatment of atherosclerosis using pharmacological agents has only been partially successful, and therefore newer therapies, either stand-alone ones or in combination with the pharmacological agents, are desirable (Figure 2). Gene therapy has been successful in human and animal models with more than 1500 clinical trials worldwide (http://www.wiley.co.uk/genetherapy/clinical/). Viral vectors appear better than nonviral vectors to deliver transgenes to target cells and tissues. Despite the setbacks to viral gene therapy due to insertional mutagenesis caused by the viral vectors or anti-viral host immune responses, better vectors with significant safety biosafety improvements have been developed Self-inactivating lentiviral vectors [245] are safer, and modifications of the viral long terminal repeats further reduce the potential of insertional mutagenesis by retro- and lentiviral vectors [287]. Even though viral integrations have been observed near transcription start sites and genes, foamy viruses have reduced preferential integration into host genes [288] in comparison to other viral systems [170, 179], and thus this class of viruses may be significantly safer than retro- or lentiviruses when used as vectors. Most of the adenoviral vectors currently being used are based on Ad5 serotype; however, since neutralizing host antibodies to the Ad5 serotype exceeds that to the Ad36 serotype in the human population [289], Ad36-derived vectors may be better for human clinical trials. Intrahepatic injection of adenoviral vectors also help in reducing the immune response and increasing transgene expression [290]. The problems arising from the preexisting host immune responses against Ad5 serotype can also be circumnavigated using vectors based on a canine adenovirus [224]. Viral vectors can also be made to specifically target the desired cell type [291–294], and this strategy should help in improving the overall efficacy of the therapy. 

Even though the first successful clinical gene therapy trial took place almost twenty years ago, work on its utility for modulating scavenger receptor function is still in its infancy (summarized in Table 3). Hepatic overexpression of scavenger receptors using viral vectors resulted in the inhibition of atherosclerosis initiation and progression [263, 265, 266] even though therapy efficacy was hampered by lack of stable long-term gene expression. Better results were obtained when a different viral vector was used [267]. A study of the long-term effect of the transgene expression *in vivo* using the other available viral vector types would be very desirable to ascertain the potential of using viral gene therapy using scavenger receptors. The splice variant isoforms of some scavenger receptors can confer protection against myocardial infarction [88], and it might be interesting to examine expression of these molecules in an *in vivo* model. The long-term effects of the expression of soluble scavenger receptors on atherosclerosis would be interesting [265–267]. Since scavenger receptor knockout models can be antiatherogenic [120], suppression of gene expression appears to be a promising strategy although effects on the host immune responses have yet to be fully understood. A dual approach where genetic manipulation of a candidate scavenger receptor is supplemented by the action of another transgene [270] combined with longer duration of transgene expression might increase therapeutic benefits in disease models. Adeno-associated viral vectors may not be very useful in the simultaneous expression of a number of transgenes from a single vector due to the limitations in their packaging capacity, but multiple transgenes can be expressed in the model system with large vectors such as HC-AdVs. Transgene expression from viral vectors such as HC-Ad and lentiviruses is relatively stable and long-lived but these vectors have been used sparingly for such studies with scavenger receptors. With the advent of gene therapy vectors with higher biosafety such as SIN-LV vectors and HC-AdV vectors, development of less immunogenic viral vectors based on nonhuman viruses [224], and suppression of transgene-specific immune responses [295] gene therapy with scavenger receptors along with other therapies might be useful in providing sustained long-term amelioration of the clinical manifestations of atherosclerosis.

## Figures and Tables

**Table 1:** The major scavenger receptor ligands and expression profiles.

Class	Scavenger receptor	Ligands	Expression profile	Involvement in CVD?
A	SR-A	AcLDL, OxLDL, *β*-amyloid, molecular chaperones, ECM, AGE, apoptotic cells, activated B-cell, bacteria	Macrophages, mast, dendritic, endothelial and smooth muscle cells	Yes—involved in OxLDL uptake by macrophages leading to foam cell formation

A	MARCO	AcLDL, OxLDL, apoptotic cells, B cells, bacteria	Macrophages, dendritic cells	No

B	SR-B	HDL, LDL, OxLDL, apoptotic cells	Monocytes/macrophages, hepatocytes and adipocytes	Reduces atherosclerosis through reverse cholesterol transport of HDL

B	CD36	AcLDL, OxLDL, HDL, LDL, VLDL, *β*-amyloid, AGE, apoptotic cells	Macrophages, platelets, adipocytes, epithelial and endothelial cells	Yes—OxLDL uptake into macrophages leading to foam cell formation

E	LOX-1	OxLDL, molecular chaperones, ECM, AGE, apoptotic cells, activated platelets, bacteria	Endothelial and smooth muscle cells, macrophages, and platelets	Yes—OxLDL uptake in endothelial cells, leads to endothelial dysfunction

F	SRECI/II	AcLDL, OxLDL, molecular chaperones, apoptotic cells	Endothelial cells and macrophages	Low levels of AcLDL uptake

G	SR-PSOX	OxLDL and bacteria	Macrophages, smooth muscle, dendritic, endothelial cells, and B- and T cells.	Yes—involved in OxLDL uptake in macrophages

H	FEEL-I/II	AcLDL, molecular chaperones, ECM, AGE, bacteria	Monocytes/macrophages, endothelial cell	No known link

SR-A: scavenger receptor class A, AcLDL: acetylated low density lipoprotein, OxLDL: oxidised low density lipoprotein, ECM: extracellular matrix, AGE: advanced glycation end products, MARCO: macrophage receptor with collagenous structure, HDL: high density lipoprotein, LDL: low density lipoprotein, VLDL: very low density lipoprotein, LOX-1: lectin-like oxidized low density lipoprotein receptor-1, FEEL-I/II: fasciclin, epidermal growth factor (EGF)-like, laminin-type EGF-like, and link domain-containing scavenger receptor-1.

**Table 2:** The advantages and disadvantages of the major gene therapy vectors currently used.

Gene therapy vector	Genetic material	Advantages	Disadvantages
Nonviral vectors	Mainly DNA	Large transgene capacity, biosafety	Low efficiency, immune response (cationic lipids and polymers), toxicity

Retro-/lentiviral vectors	RNA	Stable integration, lack of immune response, up to 10 kb cloning capacity	Insertional mutagenesis following integration is higher

Adeno-associated viral vectors	DNA	Long-term expression, site-specific integration	Immune response, small transgene capacity

First-generation adenoviral vectors	DNA	High titer, up to 8 kb of cloning capacity	Immune response and toxicity leading to shortened duration of transgene expression *in vivo *

High capacity adenoviral vectors	DNA	High titer, longevity of transgene expression, up to 36 kb cloning capacity	Immune response directed against the viral capsid

**Table 3:** List of the main viral gene therapy experiments examining the therapeutic potential of scavenger receptors.

SR used/targeted	Vector used	Outcome	References
LOX-1	FG AdV	Inhibition of the progression of atherosclerosis	Ishigaki et al. [263]
Soluble SR-A1	FG AdV	Foam cell formation inhibited	Laukkanen et al. [265]
Soluble SR-A1	FG AdV	Abrogation of the atherosclerotic lesion area	Jalkanen et al. [266]
Soluble SR-A1	AAV	Abrogation of the atherosclerotic lesion area	Jalkanen et al. [267]
SR-B1	FG AdV	Reduction of plasma HDL	Kozarsky et al. [54]
SR-B1	FG AdV	Reduction of plasma HDL	Kozarsky et al. [55]
SR-B1	FG AdV	Reduction of plasma HDL, increase of LDL	Tancevski et al. [268]
SR-B1	FG AdV	Increased biliary secretion of cholesterol	Wiersma et al. [269]
SR-B1 and Apobec 1	HC-AdV	Reduction of Apo B levels, elevation of NO	Zhong et al. [270]
SR-B1/CD36	FG AdV	SR-B1 mediated uptake of cholesterol esters higher than that by CD36. However, CD36 resulted in higher levels of Ox-LDL degradation	de Villiers et al. [271] and Sun et al. [272]
CD36	FG AdV	Increased hepatic fatty acid uptake	Koonen et al. [273]
SR-PSOX	LV	Decreased foam cell formation	Zhang et al. [120]

SR: scavenger receptor, FG AdV: first-generation adenoviral vector, AAV: adeno-associated viral vector, HC-AdV: high capacity adenoviral vector, LV: lentiviral vector, SR-A: scavenger receptor class A, LOX-1: lectin-like oxidised low density lipoprotein receptor-1, SR-B1: scavenger receptor class B 1, SR-PSOX: scavenger receptor that binds to phosphatidylserine and oxidized lipoprotein, Apobec 1: apolipoprotein B mRNA editing enzyme, catalytic polypeptide-like 1, CD36: cluster of differentiation 36, LDL: low density lipoprotein, HDL: high density lipoprotein, and NO: nitric oxide.
